# Application of eccentric training in various clinical populations: Protocol for a multi-centered pilot and feasibility study in people with low back pain and people with multiple sclerosis

**DOI:** 10.1371/journal.pone.0270875

**Published:** 2022-12-22

**Authors:** Monique Wochatz, Anne Schraplau, Tilman Engel, Mahli M. Zecher, Hadar Sharon, Yasmin Alt, Frank Mayer, Alon Kalron

**Affiliations:** 1 University of Potsdam, University Outpatient Clinic, Sports Medicine and Sports Orthopaedics, Potsdam, Germany; 2 Faculty of Health Sciences, Joint Faculty of the University of Potsdam, The Brandenburg Medical School Theodor Fontane and the Brandenburg University of Technology, Cottbus, Senftenberg, Germany; 3 Multiple Sclerosis Center, Sheba Medical Center, Tel Hashomer, Israel; 4 Department of Physical Therapy, School of Health Professions, Sackler Faculty of Medicine, Tel-Aviv University, Tel-Aviv, Israel; University of L’Aquila Department of Clinical Sciences and Applied Biotechnology: Universita degli Studi dell’Aquila Dipartimento di Scienze Cliniche Applicate e Biotecnologiche, ITALY

## Abstract

Physical activity and exercise are effective approaches in prevention and therapy of multiple diseases. Although the specific characteristics of lengthening contractions have the potential to be beneficial in many clinical conditions, eccentric training is not commonly used in clinical populations with metabolic, orthopaedic, or neurologic conditions. The purpose of this pilot study is to investigate the feasibility, functional benefits, and systemic responses of an eccentric exercise program focused on the trunk and lower extremities in people with low back pain (LBP) and multiple sclerosis (MS). A six-week eccentric training program with three weekly sessions is performed by people with LBP and MS. The program consists of ten exercises addressing strength of the trunk and lower extremities. The study follows a four-group design (N = 12 per group) in two study centers (Israel and Germany): three groups perform the eccentric training program: A) control group (healthy, asymptomatic); B) people with LBP; C) people with MS; group D (people with MS) receives standard care physiotherapy. Baseline measurements are conducted before first training, post-measurement takes place after the last session both comprise blood sampling, self-reported questionnaires, mobility, balance, and strength testing. The feasibility of the eccentric training program will be evaluated using quantitative and qualitative measures related to the study process, compliance and adherence, safety, and overall program assessment. For preliminary assessment of potential intervention effects, surrogate parameters related to mobility, postural control, muscle strength and systemic effects are assessed. The presented study will add knowledge regarding safety, feasibility, and initial effects of eccentric training in people with orthopaedic and neurological conditions. The simple exercises, that are easily modifiable in complexity and intensity, are likely beneficial to other populations. Thus, multiple applications and implementation pathways for the herein presented training program are conceivable.

**Trial registration:** DRKS00020483 (DRKS, German Clinical Trials Register; 24^th^ January 2020 –retrospectively registered; https://www.drks.de/DRKS00020483).

## 1. Introduction

Physical activity and exercise are effective approaches in prevention and therapy of a range of diseases and clinical conditions [1]. Although there is a dose-response relationship between physical activity and premature mortality as well as primary and secondary prevention of chronic diseases [2], a defined recommendation for the type and dose of exercise needed in each case, as well as its delivery, remains to be clarified [1].

A combination of aerobic and strengthening exercises, as recommended by general international guidelines [3], seems to be vital to the promotion of health. Traditional strength training, containing concentric and isometric contraction exercises has been proven to be effective in the improvement of strength, function and overall quality of life as well as disease specific symptoms for a variety of clinical conditions [4–9]. Unfortunately, eccentric training, is not frequently applied in the therapy of clinical conditions, even though specific characteristics of lengthening contractions have the potential to be beneficial for many clinical populations [10].

As a muscle lengthens under tension during eccentric contractions, it is able to generate more force than during concentric or isometric contractions [11, 12]. It is assumed that due to the higher mechanical load applied, greater muscle hypertrophy can be achieved by an eccentric exercise training [12, 13]. In addition, eccentric muscle contractions lead to structural, physiological, and metabolic responses and adaptations. For example, eccentric loads result in a lower heart rate increase and lead to a reduced cardiovascular load compared with concentric contractions [14]. Moreover, the energy demand of eccentric contractions is up to four times lower than concentric muscle contractions at the same load [15–17]. Therefore, eccentric training seems particularly suitable for patient groups with metabolic, neurologic or cardiovascular diseases and impaired exercise capacity [18] as well as other chronic diseases associated with muscle atrophy, muscle weakness and reduced physical performance.

Regardless of the benefits, eccentric exercises may initially lead to muscle damage, impaired muscle function and delayed-onset muscle soreness, especially in unaccustomed loading situations. Therefore, it could be questioned whether the use of eccentric training in the therapy of clinical at-risk populations (e.g. elderly and frail people, people with diseases of the central nervous system, people with co-morbidities) is feasible [19]. Few studies have demonstrated that eccentric training is feasible and effective in cardiorespiratory disease [20], chronic obstructive pulmonary disease [21], after cancer therapy [22] and might also be relevant in obese patients [23]. Nevertheless, the transfer into the clinical practice is pending.

To date, the majority of eccentric training programs have been focused on orthopedic indications and prevention of muscular injuries [24, 25]. Considering eccentric muscle contractions result in decelerating movements that counteract gravity [26], eccentric training could also prepare the body, and especially the trunk, for high loads that may occur repeatedly and unexpectedly during daily and work-related activities. Therefore, it is potentially beneficial in the prevention and treatment of low back pain; one of the most common clinical conditions worldwide [27]. Generally, exercise can reduce the intensity of low back pain as well as pain-dependent limitation, improve function, and prevent recurrence [28, 29]. Although there is currently no evidence indicating a preferable mode, common recommendations emphasize that exercise programs should include elements to increase muscle strength and neuromuscular control, as these factors contribute significantly to maintaining trunk stability [30, 31]. Further, the ability of the neuromuscular system to adequately compensate for load-induced dysfunction and maintaining spinal stability is highly relevant in the prevention of low back pain and its patho-anatomical consequences. Surprisingly, despite its high potential to improve muscle strength and neuromuscular capacity, eccentric training has not been thoroughly investigated as a treatment of low back pain.

People with multiple sclerosis (MS) may also benefit from the aforementioned characteristics of eccentric exercises [18]. The MS disease process produces a diversity of neuropathological changes in the central nervous system, typically affecting a wide range of neurological functions including mobility, muscle strength, coordination, and sensation. Although there is a consensus that physical activity is imperative for people with MS, to date no specific exercise mode has been proven superior over others. In this case, eccentric exercises might be a valuable approach [32, 33]. Application of high loads to improve muscle force under reduced energy cost is essential for people with MS as their energy reserves are typically low and fatigue is elicited early [34]. Furthermore, increased stimulation of the cerebral cortex [35] and motor unit recruitment patterns, as well as cross-educational effects, may improve functional capacity [11]. Moreover, sensory and muscular impairments of the lower extremity are associated with an increased risk of falls in people with MS [36]. An eccentric training program addressing the lower extremity and the trunk potentially improving gait, balance and functional mobility might therefore be beneficial to reduce the risk of falling in this population.

Only few studies have evaluated eccentric exercises in people with MS. Recently, a small pilot study demonstrated the feasibility, safety and efficacy of eccentric downhill walking in five people with MS affected by ankle contractures [37]. Additionally, eccentric strength training of the elbow flexors was proven to be feasible as well as beneficial for strength- and function-related parameters in people with MS with spasticity [38]. Moreover, because of the possible anti-inflammatory effect of eccentric training, this exercise mode seems suitable for the modulation of the immunometabolism, since MS is associated with an altered balance between pro- and anti-inflammatory cytokines [39]. In this context, Berkowitz et al demonstrated that moderate aerobic exercises induced a similar acute cytokine response in women with mild MS as age matched healthy women, considering this exercise as safe and feasible [40]. Nevertheless, since eccentric strength training may induce muscle damage and is associated with an inflammatory response [41, 42], the influence of eccentric training on cytokine levels in people with MS still warrants clarification.

Although eccentric training has a high potential in the treatment of various clinical populations, more clinical trials focusing on feasibility and efficacy are required. Therefore, the aim of this multi-center pilot study is to evaluate the feasibility, functional benefits and cytokine response of an eccentric training program focusing on trunk, and lower extremities in individuals with low back pain and MS. The decision to focus on two different clinical populations, executing an identical exercise program, was implemented in order to expand the knowledge regarding this unique exercise approach.

## 2. Methods and materials

### 2.1. Overview of the study design

**Fig 1:**
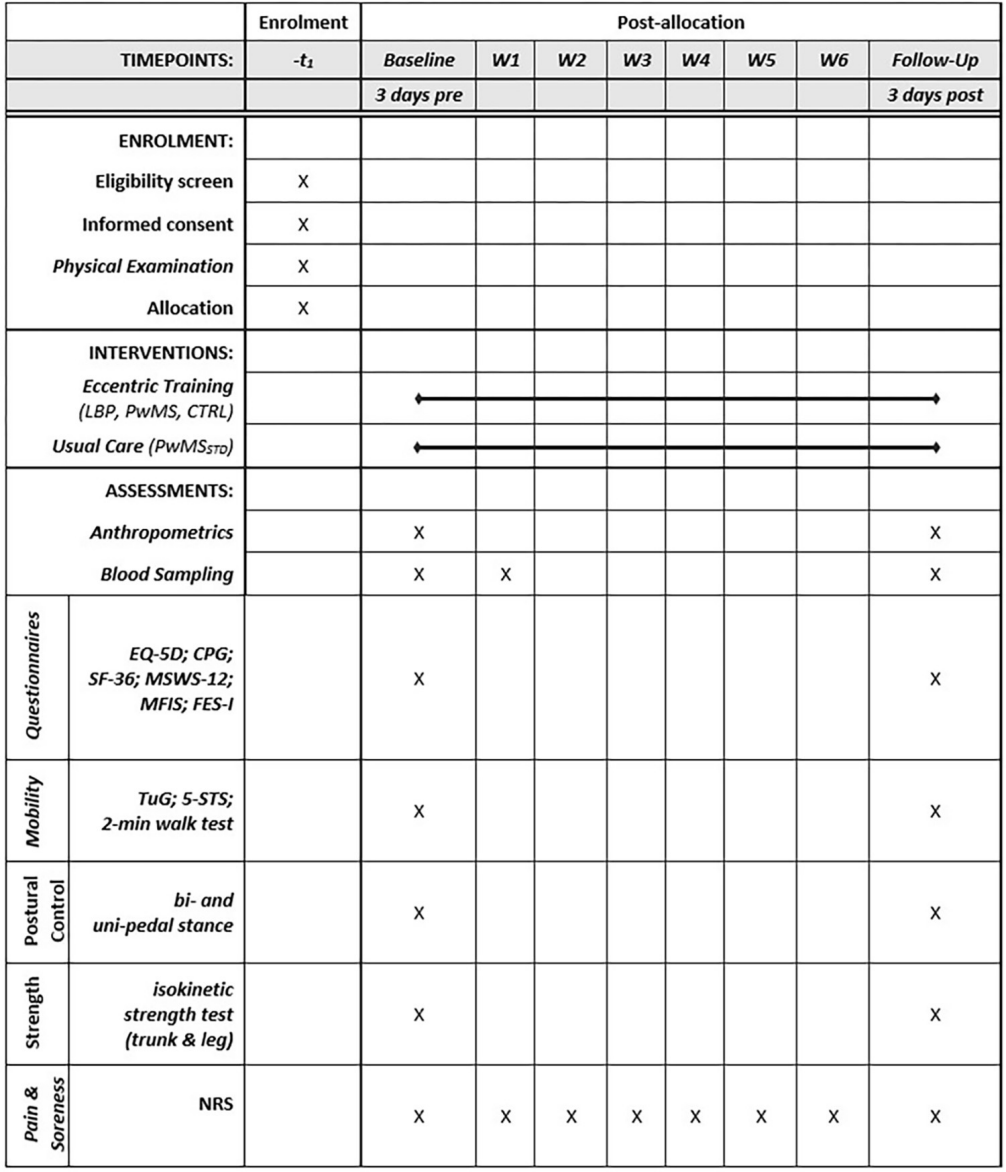
Schedule of enrollment, interventions and assessments. EQ-5D: Questionnaire developed by the EuroQol Research Foundation to assess health status; TuG: Timed Up and Go test; 5-STS: Five-times Sit-to-Stand test; CPG: Chronic Pain Grade questionnaire; SF-36: Short Form 36 health survey; MSWS-12: 12-item Multiple Sclerosis Walking Scale; MFIS: Modified Fatigue Impact Scale; FES-I: Falls Efficacy Scale–International; NRS: numeric rating scale.

**Fig 2:**
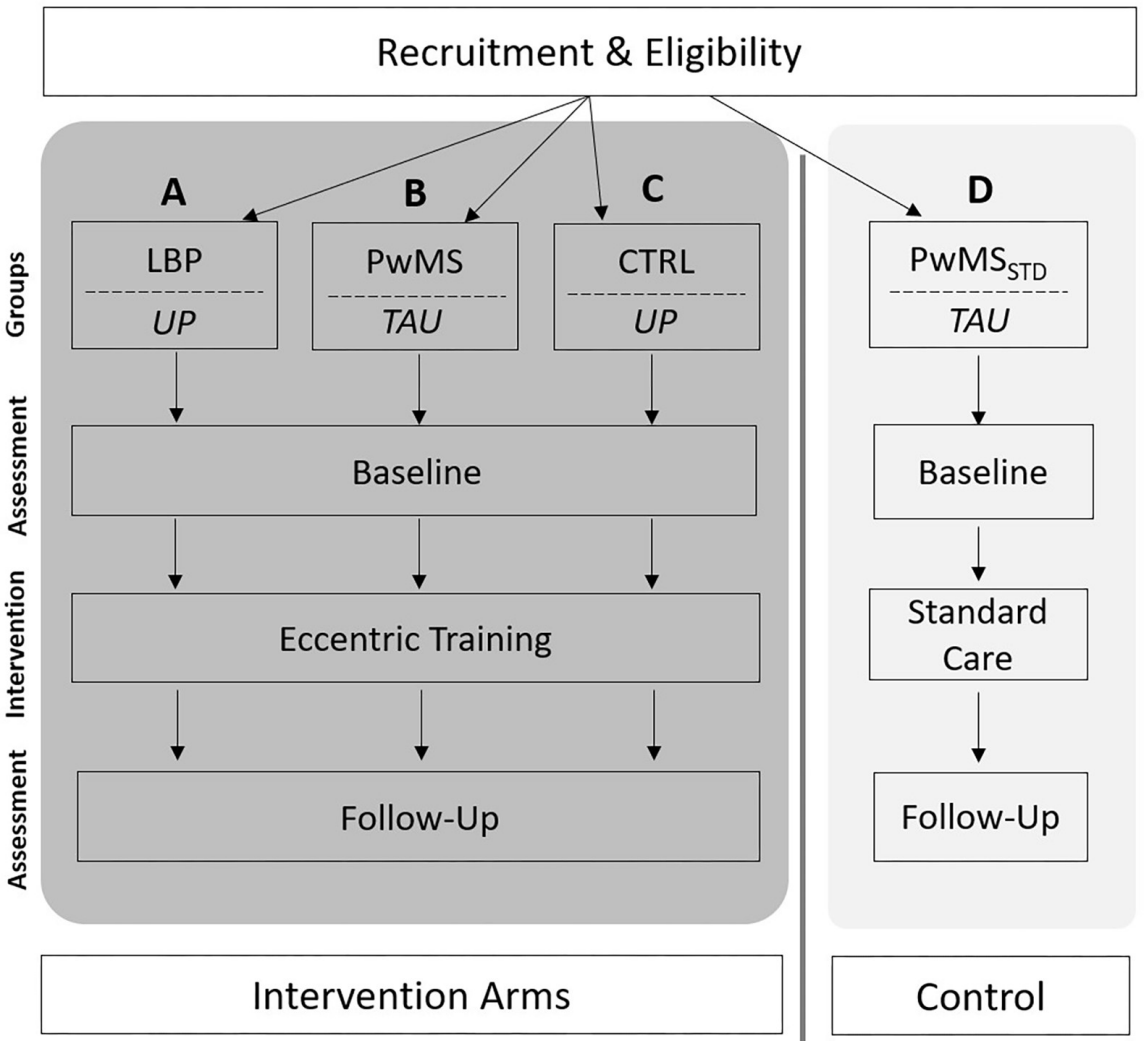
Study flow and design, showing groups that are allocated to the intervention (dark grey) and the standard care (light grey). (A) LBP: participants with low back pain–intervention group; B) PwMS: people with multiple sclerosis–intervention group; C) CTRL: asymptomatic participants–intervention group; D) PwMS_STD_: people with multiple sclerosis–standard care; UP: University of Potsdam; TAU: Tel Aviv University).

This assessor-blinded, bi-center, controlled pilot trial aims to evaluate the feasibility and benefits of a six-week eccentric training program with three weekly sessions (two center-based, one home-based), in individuals with low back pain (musculoskeletal condition) and people with MS (neurological condition). The focus is on the implementation of an eccentric training program directed to the trunk and major lower limb muscle groups. The overall approach includes a four-group bi-center design, in which three groups (A-C) perform an eccentric training program and one group receives standard care (D): (A) control group with healthy, asymptomatic people [CTRL]; B) people with low back pain [LBP]; C) people with MS [PwMS], D); people with MS who are receiving standard care physiotherapy [PwMS_STD_]. A test battery is applied to assess strength, function and patient reported outcome measures prior and following the intervention period. A schedule of enrolment, interventions and assessments is shown in **Fig 1** and the study design and flow in **Fig 2**. The study has been approved by the Ethics Committee of the University of Potsdam, Germany (ref. No. 44/2019) and the Institutional Review Board of the Sheba Medical Center, Tel-Hashomer, Israel (Nr. SMC-6288-19). Any kind of study amendment will be approved by the above-mentioned review boards prior to implementation and will be reported to the trial registry. The study is conducted and performed in agreement with the Declaration of Helsinki. This trial was registered under www.drks.de and the trial number is DRKS00020483 (DRKS, German Clinical Trials Register; 24^th^ January 2020). This paper is further prepared in accordance with the “Standard Protocol Items: Recommendations for Interventional Trials (SPIRIT)” guidelines (see **S1 Table**).

### 2.2. Participants

The study is performed at two study centers, one in Germany and one in Israel. Participants of the CTRL and LBP group are recruited via the University Outpatient Clinic of the University of Potsdam, Germany. Participants with MS are recruited within the Multiple Sclerosis Center, Sheba Medical Center, Tel Hashomer, Israel. For the rational of a feasibility study a sample size of 12 participants per group is intended [43]. Participants (all sexes) aged between 18 and 65 years are included into this study. Participants eligible for CTRL group are healthy asymptomatic adults, with absence of any acute or chronic LBP. Asymptomatic participants with acute infections, pregnancy, or structural changes of the spine with or without neurological signs contra-indicating physical activities are excluded. Participants eligible for the LBP group need to report a minimum of 3 low back pain episodes within the last 12 month (not exclusively within the last 3 month) and report an average pain score of 3 or above on the numeric rating scale (NRS) within the last 3 months (NRS: numeric rating scale; 0: no pain; 10: worst imaginable pain). People with acute low back pain which occurred for the first time within the last 7 days, acute infections, pregnancy, or structural changes of the spine, with or without neurological signs contra-indicating physical activities are excluded. Participants eligible for PwMS and PwMS_STD_ group need a neurologist-confirmed diagnosis of definite MS according to the revised McDonald criteria, a score of <4.5 on the Expanded Disability Status Scale (EDSS), the ability to walk an equivalent of at least 500m without a walking aid and to be relapse-free for at least 90 days prior to testing. People with MS with cardiovascular disorders, respiratory diseases, the use of steroids or fampridine or other conditions contra-indicating physical activities are excluded.

### 2.3 Intervention programs

#### 2.3.1 Eccentric intervention

The program contains ten exercises addressing strength of the trunk and lower extremity, five exercises each for each body region (see **S1 Fig**). During execution of exercises, patients are instructed to emphasize the eccentric phase by performing the movement slowly and in a controlled manner against gravity. The program focuses on body weight exercises, complemented by resistance bands and small weights. Center-based training sessions are supervised by a team of experienced sport- and physiotherapists. Over the course of the intervention period (18 sessions total) each exercise is performed at least at six training sessions, ensuring a balanced and standardized exercise selection for all participants. Each training session comprises four to six individual exercises, which are performed for two to three sets with 10 to 15 repetitions each set. Intensity is controlled by a self-reported rating of perceived exertion (RPE) via the Borg Scale [44, 45]. Adequate training intensity is set by an RPE score between 13 and 17. Additional modifications regarding exercise complexity and intensity are made individually by controlling the range of motion, lever arms, motion velocity and use of additional resistance, respectively. For each training session RPE, pain intensity as well as muscle soreness are assessed (see **S2 Fig**). Overall weekly physical activity as well as all scores related to the eccentric program are documented and stored in a training log.

#### 2.3.2 Usual care for people with MS

The standardized physical therapy comprises twelve 40-45-minute individualized face to face physiotherapy sessions, over a 6-week period, plus an individualized 30-minute daily home exercise program. The standardized program of physiotherapy exercises aims at improving trunk and pelvic stability, lower limb muscle length, strength, balance and control of movement, in accordance with the Bobath concept [46]. This exercise program is reflective of the general exercises typically undertaken within routine clinical practice in people with MS.

#### 2.3.3 Adverse events

Adverse events like substantial increases in pain levels or any kind of symptom worsening are documented by either trained sport- and physiotherapist or the study team for events that occur during the intervention as well as during the baseline and follow-up assessments. If participants experience adverse side effects of the interventions or the test battery, consultation with a physician is sought to discuss necessary modifications or an early termination of the study.

### 2.4. Assessments/Procedure

#### 2.4.1 Experimental procedure

Potential participants learn of this study through personal contact, e-mails and printed advertisements posted at the two centers. The study team’s contact details are provided to enable interested participants to further enquire and participate in the study. Interested participants are provided with an information pack describing the study, supported by verbal information. They are screened for eligibility according to the pre-specified inclusion and exclusion criteria at the above-mentioned study sites. With inclusion criteria met, written informed consent of the participants are obtained. Group assignment is randomly performed where applicable by the principal investigators based on a generated list matched by age and gender. The randomization list is kept concealed (password protected, exclusively accessed by principal investigator) until intervention assignment. Only data analysts remain blinded to group allocation. Prior to the baseline measurement, participants undergo a physical examination (self-reported anamneses: verified diagnosis; current pain level, complaints and impairments; musculoskeletal/orthopedic examination) by a physician to rule out contraindications that restrict or prevent participation in regard to testing and exercise intervention. Baseline measurements (M1) are conducted three days before the first training session, comprising: (1) blood sampling, (2) self-reported questionnaires, (3) mobility, (4) balance and (5) strength testing. All participants, irrespective of group allocation, perform the test battery. Additional blood sampling is conducted immediately before and three days after the first training session in order to assess the acute reaction to the initial training bout. The follow-up measurement (M2) is conducted three days after the last training session. The follow up assessment session includes tests identical to those collected at baseline, with the addition of participant rating scores of the intervention program (5-point Likert-Scale regarding exercise selection, time effort and required equipment).

#### 2.4.2 Common/Joint assessments

Venous blood samples are taken from an antecubital forearm vein using disposable needles and vacutainers. Blood samples are centrifuged and serum samples frozen and stored (in aliquots at -80°C) for the analysis of markers representing the muscle metabolism (creatine kinase, myoglobin) [42] and inflammatory reactions (C-reactive protein, interleukins (IL-6, IL-8, IL-10, IL-1β, IL-1ra, IL-4), tumor necrosis factor-alpha) [41] in response to eccentric exercises and standard physiotherapy care for PwMS.

The general health status is assessed with the *EQ-5D* questionnaire (Version: EQ-5D-5L) which consists of descriptive questions addressing the dimension mobility, self-care, usual activities, pain/discomfort and anxiety/depression and a visual analog scale (VAS; 0–100) for a subjective rating of their own overall current health. The health state for each dimension is thereby represented by a number of 1–5 indicating no problem, slight problems, moderate problems, severe problems or unable to/extreme problems [47, 48].

The “Timed Up and Go test” (TuG) and “Five-times-Sit-to-Stand test” (5-STS) are utilized to assess functional capacity according to strength, agility and dynamic balance during conditions of body transition (sitting to standing/standing to sitting), walking and changing directions [49–51]. The time (s) to completion of the movement task is considered. Additionally, postural control is assessed during static balance tasks incorporating bi-pedal (eyes-open and eyes-closed) and uni-pedal stance over a time period of 30s [52, 53]. Measurements of the postural sway are based on the trace of the center of pressure (COP) excursion (mm) assessed via a custom-made balance board (Wii Balance Board, Nintendo, Kyoto, Japan; modified by CSMi Computer Sports Medicine Inc., Stoughton, MA, USA). The standardized procedure for the conduction of the functional tests includes a prior demonstration by the investigator followed by a practice trial of the participant. Afterwards two performed trials are assessed.

Force capacity of the knee flexors and extensors are assessed via isokinetic dynamometry (University Outpatient Clinic—Potsdam: Con-Trex, MJ, Physiomed AG Germany; Sheba Medical Center–Tel Aviv: CSMi Computer Sports Medicine Inc., Stoughton, MA, USA) [54–56]. The isokinetic strength test of maximum voluntary contractions (MVC) is performed within a predefined range of motion of 70° (15° to 85° knee flexion) and at a motion velocity of 60°/s. Flexion and extension are performed in an alternating manner. The isokinetic protocol consists of a warm-up (10 repetitions; 50% of MVC, concentric) followed by five repetitions of concentric MVCs and a familiarization trial of five eccentric flexion/extension movements (50% MVC) followed by five repetitions of eccentric MVCs. Trials are separated by a one-minute break. For the evaluation of maximum force capacity, peak torque (Nm) and work (J) of concentric and eccentric knee flexion and extension will be considered.

#### 2.4.3 Population-specific assessments

For LBP and CTRL, pain severity in regard to low back pain is assessed by the “Chronic Pain Grade questionnaire” considering the subscales of characteristic pain intensity, pain related disability and the overall pain grade classification [57, 58].

For PwMS and PwMS_STD_ the following questionnaires will be used in addition to the common assessments mentioned above: SF 36 quality of life in MS (SF-36), 12 item Multiple Sclerosis Walking Scale (MSWS-12), Modified Fatigue Impact Scale (MFIS), Falls Efficacy Scale International (FES-I). The MSWS-12 is currently the most widely, qualitative, patient-reported outcome measure assessing the patients’ perception of the impact of MS on walking ability. The MFIS is a modified form of the Fatigue Impact Scale based on items derived from interviews with MS patients concerning how fatigue impacts their lives. This instrument provides an assessment of the effects of fatigue in terms of physical, cognitive and psychosocial functioning. The SF-36 is one of the most widely used generic measures of health-related quality of life and has been shown to discriminate between subjects with different chronic conditions and between subjects with different severity levels of the same disease. This instrument addresses health concepts that are relevant to MS patients from the patient’s perspective. The SF-36 generates eight subscales and two summary scores. The eight subscales are: physical functioning, role limitations due to physical problems, bodily pain, general health perceptions, vitality, social functioning, role-limitations due to emotional problems, and mental health. The two summary scores are the physical component summary and the mental component summary. The FES-I is a self-administered questionnaire designed to assess fear of falling in different clinical populations. The questionnaire comprises 16 items, including a range of functional activities. Individuals are instructed to rate each activity on a 4-point Likert scale, depending on how concerned they are that they might fall if they did this activity, regardless of whether they actually perform it.

For LBP and CTRL force capacity is assessed additionally for the trunk flexors and extensors. The instrumentation as well as the protocol corresponds with the strength testing at the knee. Trunk motion is set to a range of 55° (45° flexion to 10° extension) [56].

### 2.5 Outcome parameters

#### 2.5.1 Primary outcomes

The feasibility of the eccentric training program in people with low back pain and MS is evaluated by means of quantitative and qualitative measures in regard to the practical aspects of study conduction, the measurement setup and procedure and the performance of the intervention program. To evaluate the study process, recruitment rate, retention and drop-out rates will be assessed. Compliance and adherence of the participants to the eccentric exercise program are based on the training log, detailing a) how many sessions are done per week, b) which exercises are performed, c) at which intensity is trained (RPE via Borg Scale) and d) whether exercises are modified. Adverse events throughout the intervention period are documented to evaluate the safety of the program. Additionally, a subjective rating (5-point Likert-Scale) of the participants on the question whether the exercise program can be realized in a good manner regarding the exercise selection, the required time and materials is used to evaluate the feasibility of the implementation.

#### 2.5.2 Secondary outcomes

Besides the primary outcomes on study feasibility, additional surrogate parameters, such as preliminary assessments of possible intervention effects on overall health status, mobility, and postural control as well as muscle strength, are derived. Changes in the surrogates from baseline to follow-up assessment are considered and form the basis for future multi-center investigations on the efficacy of the eccentric training program. Further, the amount and type of missing data in the specific population groups are evaluated to identify possible bias during the measurement and intervention procedure. All outcome parameters are listed in **Table 1**.

**Table 1:** Feasibility and surrogate outcomes of the pilot intervention study.

Feasibility Outcomes	Surrogate Outcomes
**Study process:** • recruitment rate • retention rate • drop-out rate **Compliance and adherence:** • performed sessions [N] • adaptations to exercise selection and exercise modification [comments] • exercise intensity: RPE [Borg scale; 6–20] **Safety:** • adverse events [N] • pain [NRS, 0–10] • muscle soreness [NRS, 0–10] **Program evaluation:** • exercises, time, material [5-point Likert-scale]	**Questionnaires:** • EQ-5D • CPG (only LBP) • SF-36, MSWS-12, MFIS, FES-I (only PwMS) **Mobility:** • TuG [s]; 5-STS [s] • 2min-walk test [m] (only PwMS) **Postural Control:** • COP sway length [mm] for bi- & uni-pedal stance **Strength:** • peak torque [Nm] knee flex/ext • peak torque [Nm] trunk flex/ext (only LBP) **Blood Samples:** • creatine kinase • myoglobin • C-reactive protein • interleukins (IL-6, IL-8, IL-10, IL-1β, IL-1ra, IL-4) • tumor necrosis factor-alpha

RPE: rating of perceived exertion; NRS: numeric rating scale; EQ-5D: Questionnaire developed by the EuroQol Research Foundation to assess health status; TuG: Timed Up and Go test; 5-STS: Five-times Sit-to-Stand test; CPG: Chronic Pain Grade questionnaire; SF-36: Short Form 36 Health Survey; MSWS-12: 12-item Multiple Sclerosis Walking Scale; MFIS: Modified Fatigue Impact Scale; FES-1: Falls Efficacy Scale–International.

### 2.6 Dta management and statistics

Identifying data of the participants will be recorded once after their inclusion into the study and kept separately from all other study documents. The collected data will be pseudonymized. Documentation and storage of the measurement data takes place on password-protected, department/clinic-internal computers as well as on a web-based documentation form in accordance with the applicable data protection regulations of both countries. All study documents are locked at all times in office spaces only accessible to study team members.

Data of feasibility and surrogate outcomes are analyzed descriptively considering measures of frequency, central tendency, and distribution. The eccentric intervention program will be considered feasible when 70% of the participants adhered to two-third of the training sessions and performed the second measurement after the intervention period [59–62]. To allow conclusions about the potential efficacy of the intervention program only participants that adhered to prescriptions of the exercise program are included in the comparison of baseline and follow-up assessments. Prior to any data analysis plausibility and range checks will be carried out. A per protocol approach is used in the pilot investigation even though an intention-to-treat approach will be preferred for future investigations on the effectiveness of eccentric exercise programs. Possible intervention effects on: patient reported outcomes, mobility, postural control, strength, and muscle-metabolism as well as inflammatory markers are tested by a two-way repeated measures ANOVA (within factor: time, between factor: group allocation). If the variance analysis indicates interaction effects, post hoc testing with respective Bonferroni correction is applied to identify group differences depending on measurement time points. Effect sizes associated with univariate F-statistics will be expressed as eta-squared (η^2^) and effect sizes based on a difference in mean scores will be expressed as Cohen’s d. Study results are planned to be communicated via peer-reviewed international journal publications.

## 3. Discussion

It is of overall interest to evaluate whether eccentric exercises can be applied in clinical populations that would potentially benefit from eccentric loading specific effects. Evidence is slowly accumulating regarding the use of eccentric exercises in for example clinical populations with cardiorespiratory disease [20], chronic obstructive pulmonary disease [21], cancer [22] and obese patients [23]. However, studies are rare for orthopedic and neurological conditions even though those populations would potentially benefit from eccentric strengthening exercises. Therefore, it is the purpose of the study presented in this protocol to investigate the feasibility of a six-week eccentric exercise program in people with low back pain and MS. It might thereby form the foundation for future studies assessing the efficacy of eccentric strengthening exercises and the facilitation of eccentric training modalities in the therapy of orthopedic and neurological disorders. Often a safe application of high mechanical loads is questioned in those populations. The present study will add knowledge on the safety, practicability and initial effects regarding strength, postural control, function and mobility as well as inflammatory reactions of an eccentric strengthening program with moderate to heavy training intensities. Further, the adherence to exercise programs is vital for their effectiveness. Surveys show that a large proportion of adults do not meet recommendations on physical activity which might be due to individual limitations, such as low baseline level and inexperience with intense physical exertion, limited physical capacity or impairment and disability due to medical circumstances, limited availability of sports facilities and equipment, or economic constraints [63, 64]. The adherence to exercise therapy programs is critical for clinical outcomes in people with low back pain [65] and MS [66] as well as in patients with other clinical conditions [67]. Thus, training programs need to be targeted, efficient, and fit into daily life in order to increase adherence in the prevention and therapy of diseases [68]. The eccentric exercise program presented in this protocol may address some of the afore-mentioned obstacles and supports the adherence and compliance as the program consists of simple exercises that can be easily modified in their complexity and intensity according to the individual physical capacity of the patient. Moreover, exercises can be performed without heavy equipment (only small weights and resistance bands) which allows a conduction in both, clinical settings and home environments. Furthermore, the program presents a collection of exercises addressing different muscle groups of the trunk and lower extremity facilitating a variety during the process of exercise progression. Documentation of adherence and compliance in accordance with initial intervention effects within the present study will allow first insights into dose-response relationships and may be helpful during the development of future study programs. Even though this eccentric program is designed for the treatment of people with low back pain and MS, it contains basic exercises for the trunk and the lower extremity which may be as well beneficial for other populations, due to the unspecific nature of the selected exercises. However, the generalizability of findings especially for populations with increased impairments seem limited as only participants with mild low back pain and MS (<4.5 EDSS) are recruited in this study.

Another decisive aspect for the implementation of exercise programs is the practicability and transferability in the everyday life of the patients. Commonly, exercise therapy programs are guided by a physiotherapist or sport therapist in a center-based setting. Especially in rural areas with lower accessibility of health care providers due to limited infrastructure, larger distances and reduced mobility of older people and people with physical limitations, home based programs could be a good addition or alternative to center-based approaches [69, 70]. Moreover, home-based programs could enable the continuation of an exercise therapy after intensive inpatient rehabilitation. Because the exercises selected for the presented program are simple, require little equipment, and can be performed independently after brief instruction by a therapist, the program would be also suitable for home-based use or telerehabilitation approaches. Adjusting the difficulty of the exercises and discussing the continuation of the program could be done in regular phone calls or video conferences with a supervising therapist. Accordingly, there could be multiple areas of application and delivery pathways for the exercise program presented herein suitable in different settings and clinical populations.

## Supporting information

S1 Table SPIRIT checklist. (PDF)

S1 Fig Extraction of training diary. Ten exercises (5 exercises with focus of trunk strength (A-E) and 5 exercises with focus of leg strength (F-J)) and their specific movement descriptions with starting position, end position and transition back to starting position. Additionally, options to modify the exercise and increase or decrease the intensity/complexity are depicted.
(PDF)

S2 Fig Extraction of training log. Exemplary weekly documentation sheet of training sessions, selected exercises, assessment of pain and muscle soreness and additional exercise sessions apart from the intervention. The comment box allows participants to explain any deviation from the exercise selection and prescription.
(PDF)

S1 File (PDF)

S2 File (PDF)

S3 File (PDF)

## List of abbreviations

MS: multiple sclerosis
CTRL: control group
LBP: low back pain group
PwMS: people with multiple sclerosis group
PwMS_STD_: people with multiple sclerosis who receive standard physiotherapy care
UP: University Potsdam
TAU: Tel Aviv University
NRS: numeric rating scale
EDSS: Expanded Disability Status Scale
RPE: rating of perceived exertion
M1: baseline measurement
M2: follow-up measurement
EQ-5D: Questionnaire developed by the EuroQol Research Foundation to assess health status
VAS: visual analog scale
TuG: Timed Up and Go test
5-STS: “Five-times-Sit-to-Stand test”
COP: center of pressure
MVC: maximum voluntary contraction
SF-36: Short Form 36 quality of life in MS
MSWS-12: 12 item Multiple Sclerosis Walking Scale
MFIS: Modified Fatigue Impact Scale
FES-I: Falls Efficacy Scale International
