# Correction: Application of eccentric training in various clinical populations: Protocol for a multi-centered pilot and feasibility study in people with low back pain and people with multiple sclerosis

**DOI:** 10.1371/journal.pone.0293225

**Published:** 2023-10-17

**Authors:** Monique Wochatz, Anne Schraplau, Tilman Engel, Mahli M. Zecher, Hadar Sharon, Yasmin Alt, Frank Mayer, Alon Kalron

There are errors in the Funding section. The correct Funding statement is: Funded by the Deutsche Forschungsgemeinschaft (DFG, German Research Foundation)–Projektnummer 491466077.
