# Supplementary material for: Application of eccentric training in various clinical populations: Protocol for a multi-centered pilot and feasibility study in people with low back pain and people with multiple sclerosis
Source: PLoS One. 2022 Dec 22;17(12):e0270875. doi: 10.1371/journal.pone.0270875 (PMC9779041; doi:10.1371/journal.pone.0270875)

Exercise Overview

(A) Trunk extension

Movement description

- Lowering of upper body/trunk
- keep upper body/spine straight over the course of movement
- upward movement assisted by arms: push trunk up with arms to get back to starting position

Starting Position

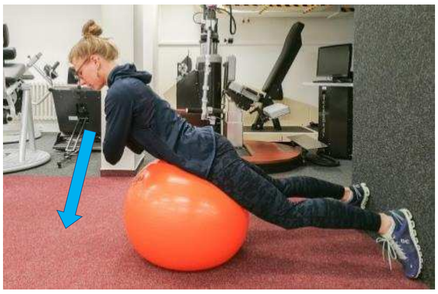

End Position

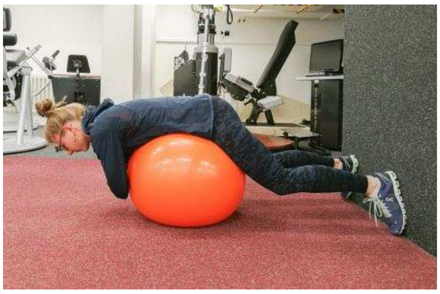

Get back to Starting Position

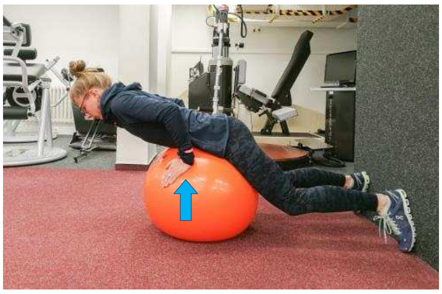

Adjustment of intensity

Decreased intensity ↓

reduction of movement amplitude

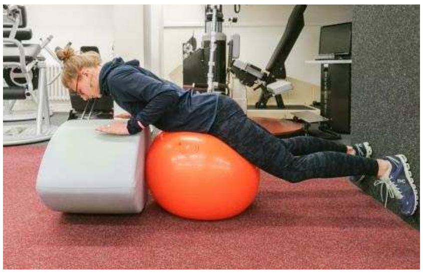

Increased intensity↑

arms extended overhead, add. load applied by weights, held by arms

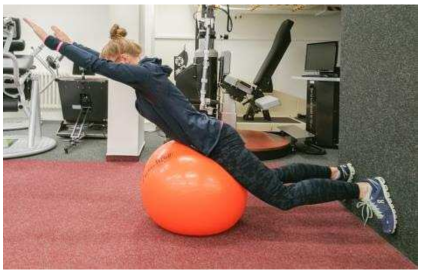

(B) Crunches

Movement description

- Lowering of upper body/trunk
- upward movement assisted by arms: push trunk up with arms to get back to starting position

Starting Position

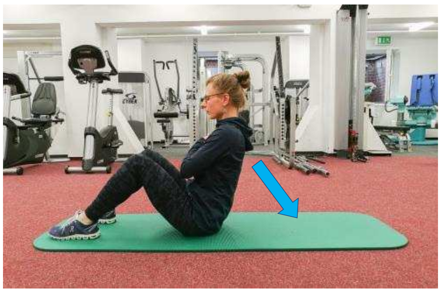

End Position

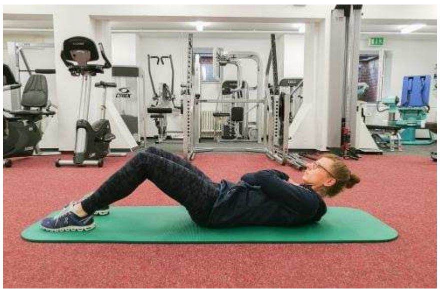

Get back to Starting Position

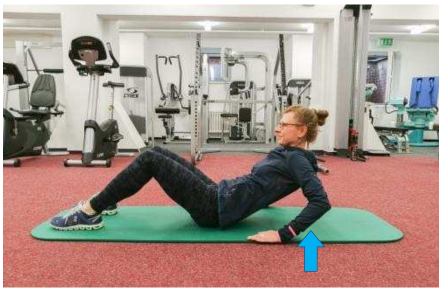

Adjustment of intensity

Decreased intensity ↓

reduction of movement amplitude

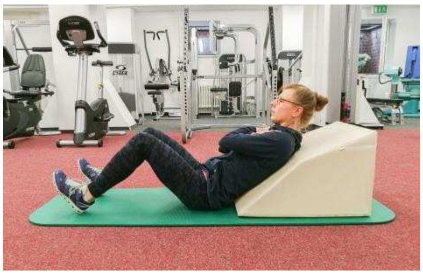

Increased intensity ↑

arms extended overhead,

add. load applied by weights held by arms in front of chest or overhead

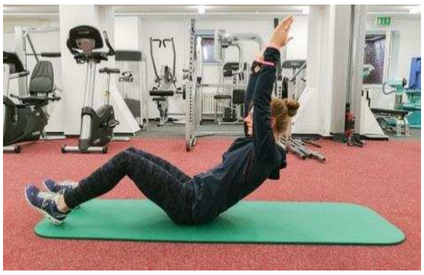

Exercise Overview

(C) Lateral Flexion

Movement description

- Lowering of upper body/trunk
- keep upper body/spine straight over the course of movement
- upward movement assisted by arms: push trunk up with arms to get back to starting position

Starting Position

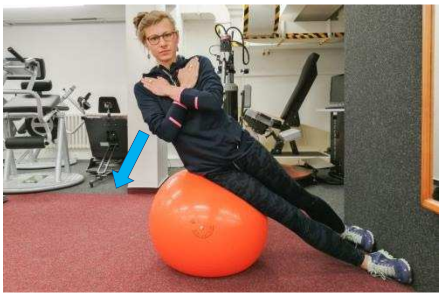

End Position

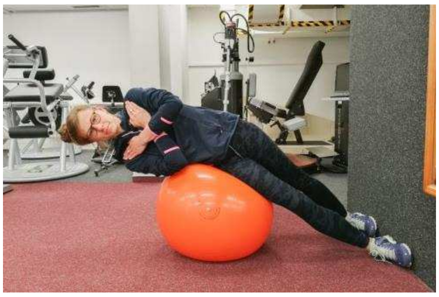

Get back to Starting Position

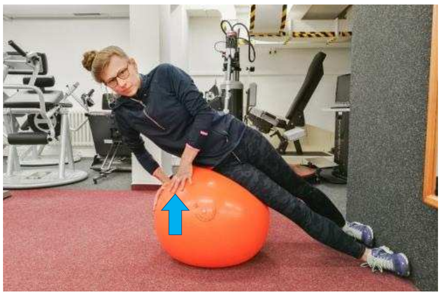

Adjustment of intensity

Decreased intensity ↓

reduction of movement amplitude

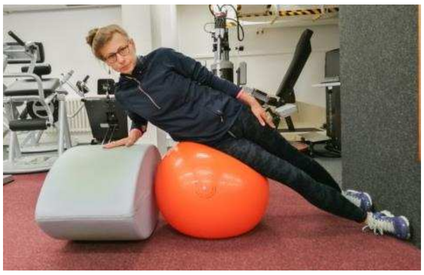

Increased intensity ↑

arms extended overhead

additional load by weights held by arms

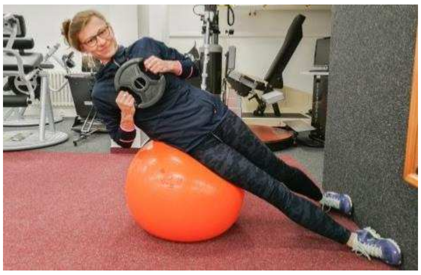

(D) Trunk Rotation

Movement description

- rotation of upper body while legs & hip are kept stable (keep upper body straight)
- flexion of arms to chest + rotation of the trunk to get back to starting position
-

Starting Position

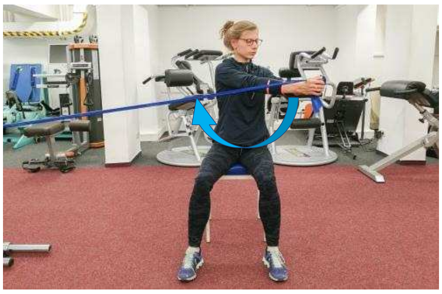

End Position

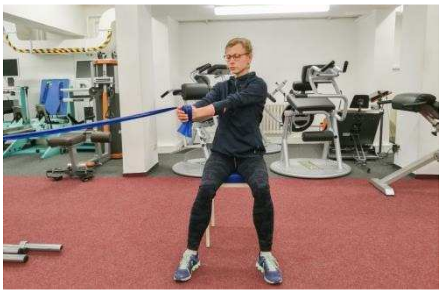

Get back to Starting Position

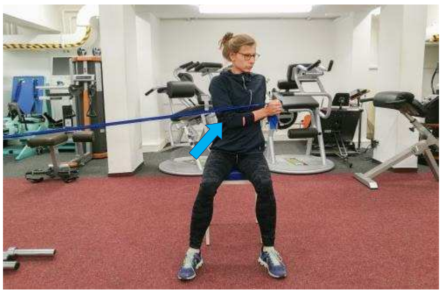

Adjustment of intensity

Decreased intensity ↓

lying position with legs flexed at knee and hip joint:

- move legs from one side to another
- extend legs and rotate back to starting position

Start Position

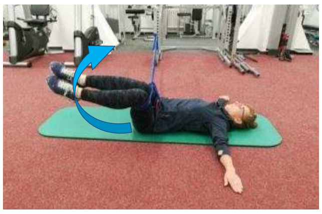

End Position

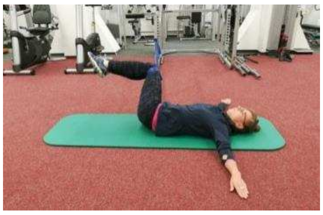

Get back to Start Position

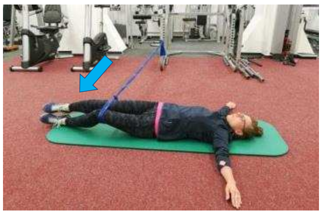

Exercise Overview

(E) Bridging

Movement description

- lowering of bottom while one leg is extended (keep hip straight/no tilting)
- push hip up with both legs on the ground to get back to starting position

Starting Position

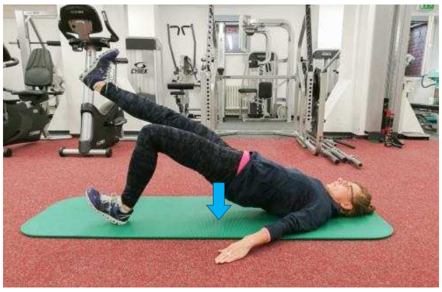

End Position

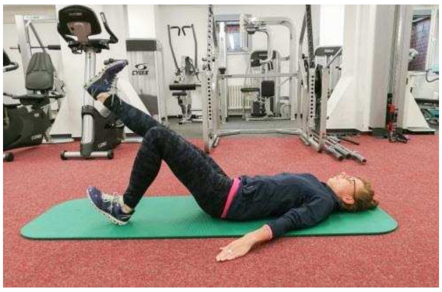

Get back to Starting Position

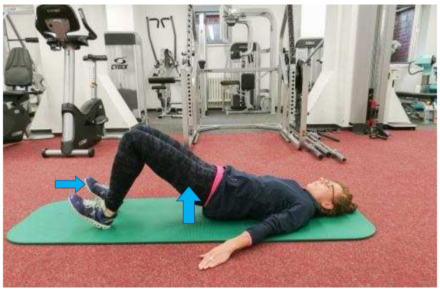

Adjustment of intensity

Decreased intensity ↓

reduction of movement amplitude

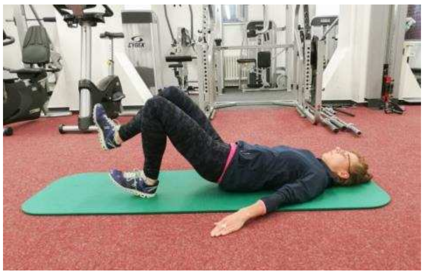

Increased intensity↑

shoulder girdle elevated (e.g. on bench),

add. load (weights/ resistance band at hip)

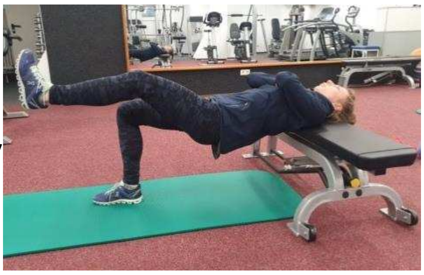

(F) Hip Extension

Movement description

- move leg from hip extension to flexion (keep hip straight/no tilting)
- move flexed leg (hip and knee) to hip extension and then extend knee to get back to starting position

Starting Position

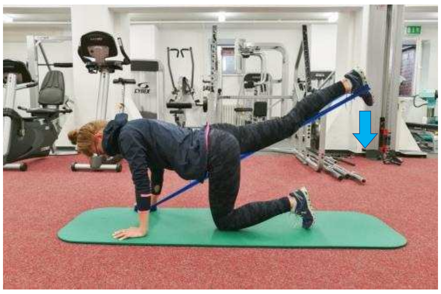

End Position

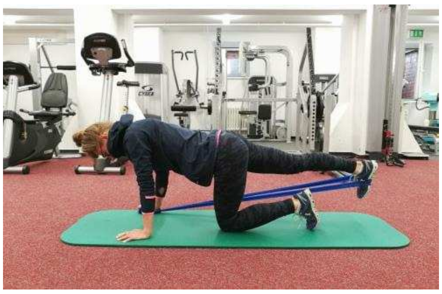

Get back to Starting Position

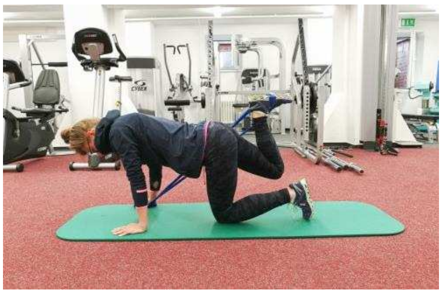

Adjustment of intensity

Decreased intensity ↓

reduction of movement amplitude,

decreased load

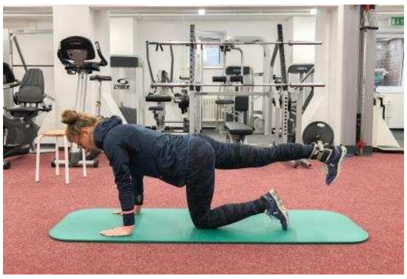

Increased intensity↑

standing position: movement from hip extension to hip flexion, load applied by pulley/resistance band

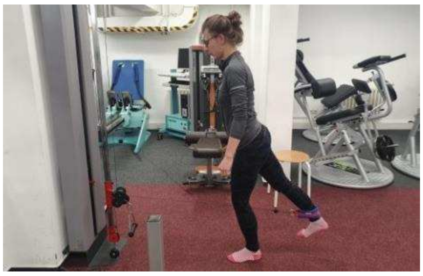

Exercise Overview

(G) Squats

Movement description

- during one-leg stands move standing leg from extension to flexion (move bottom backwards)
- put both legs on the ground and push body up to straight standing to get back to starting position

Starting Position

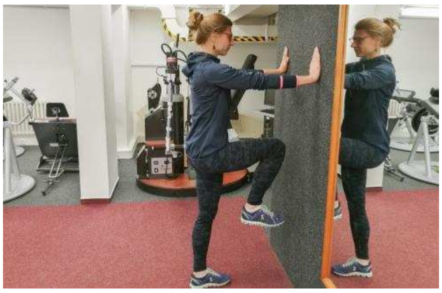

End Position

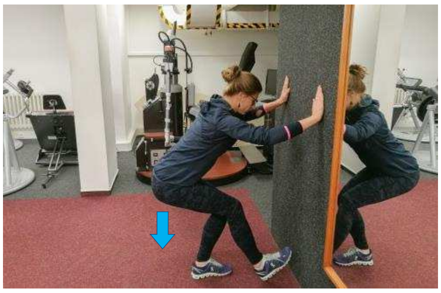

Get back to Starting Position

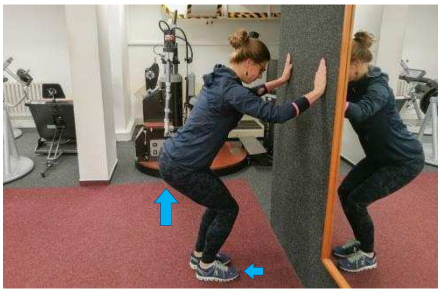

Adjustment of intensity

Decreased intensity ↓

reduction of movement amplitude

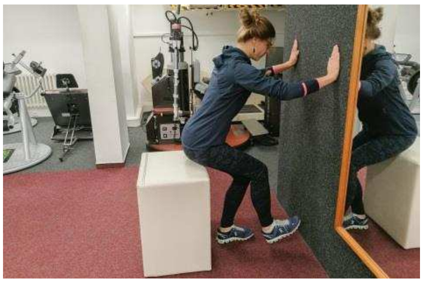

Increased intensity ↑

additional load applied by weight (e.g. backpack, shoulder girdle, hands)

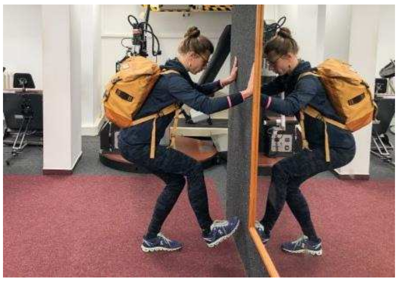

(H) Hip Abduction

Movement description

- move upper leg from abduction to adduction
- flexion in hip & knee joint followed by abduction of leg & extension in hip & knee to get back to starting position

Starting Position

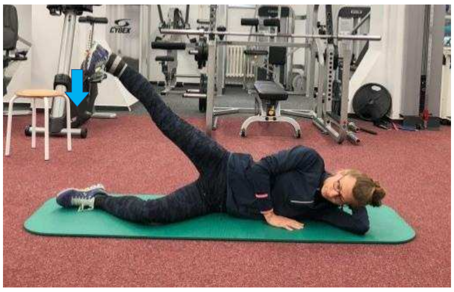

End Position

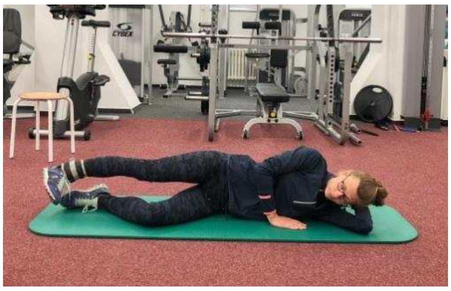

Get back to Starting Position

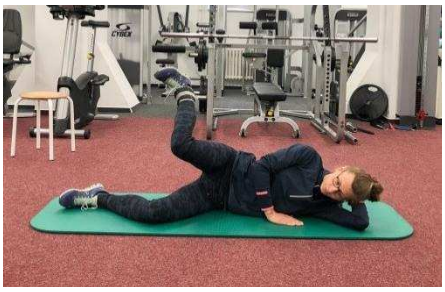

Adjustment of intensity

Decreased intensity ↓

reduction of movement amplitude

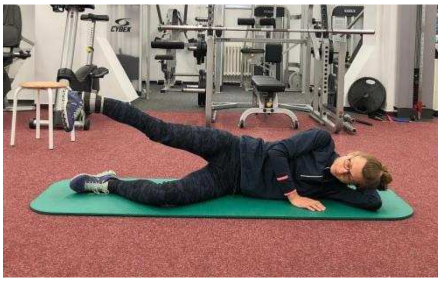

Increased intensity ↑

standing position: movement from hip abduction to hip adduction,

load applied by pulley/resistance band

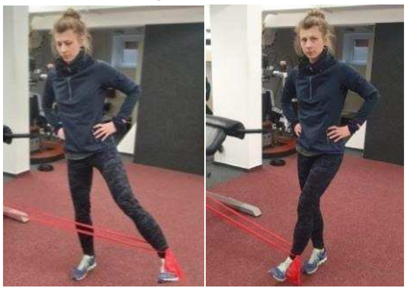

Exercise Overview

(I) Hip Rotation

Movement description

- in side lying position move upper knee down to lower knee (keep upper body straight and hip vertical)
- assist with hand to move upper leg back to starting position

Starting Position

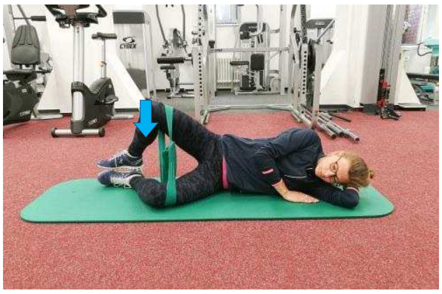

End Position

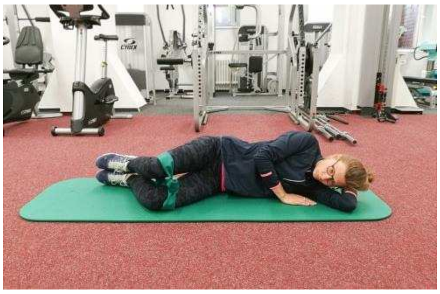

Get back to Starting Position

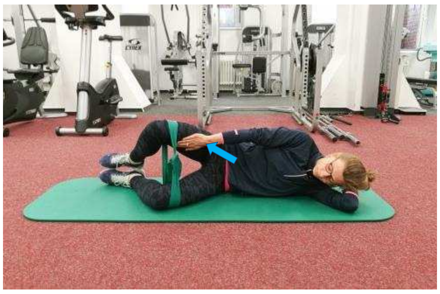

Adjustment of intensity

Decreased intensity ↓

reduction of movement amplitude,

decreased load

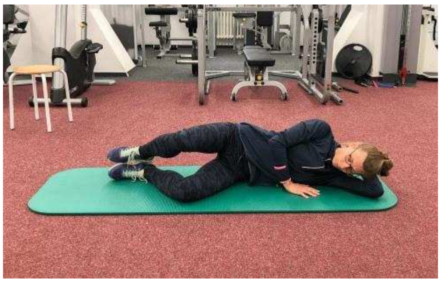

Increased intensity↑

standing position: movement from hip external rotation to hip internal rotation, load applied by resistance band

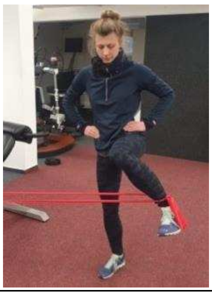

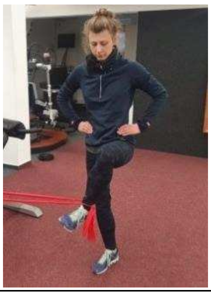

(J) Leg Curl

Movement description

- while legs are fixated (by therapist/tuck under object) lowering of upper body/trunk
- push trunk up with arms to get back to starting position

Starting Position

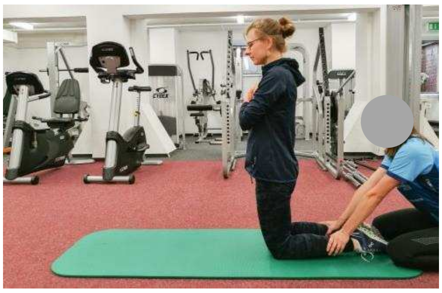

End Position

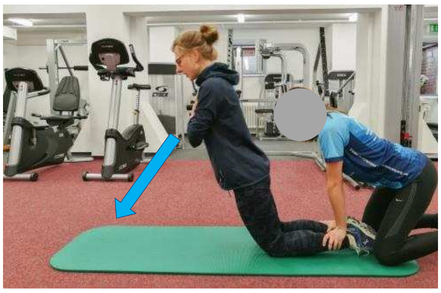

Get back to Starting Position

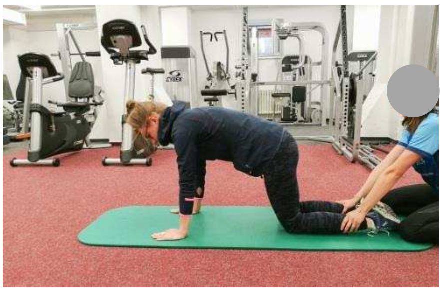

Adjustment of intensity

Decreased intensity ↓

reduction of movement amplitude

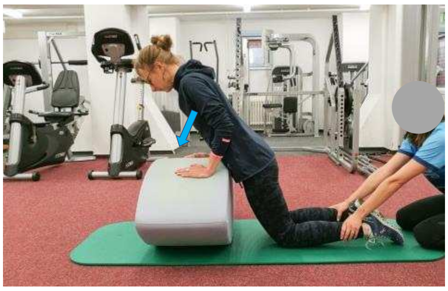

Increased intensity↑

lying position with legs on swiss ball: extension in knee to move ball away from body, both legs bring ball back to bottom

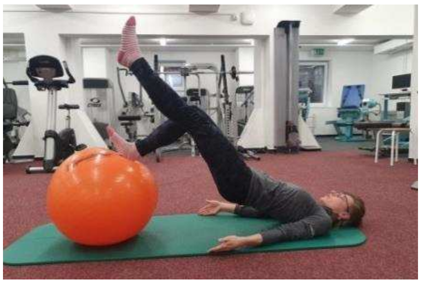

Supplement: S1 Fig — Ten exercises (5 exercises with focus of trunk strength (A-E) and 5 exercises with focus of leg strength (F-J)) and their specific movement descriptions with starting position, end position and transition back to starting position. Additionally, options to modify the exercise and increase or decrease the intensity/complexity are depicted. (PDF) [file pone.0270875.s002.pdf]
