# Supplementary material for: Application of eccentric training in various clinical populations: Protocol for a multi-centered pilot and feasibility study in people with low back pain and people with multiple sclerosis
Source: PLoS One. 2022 Dec 22;17(12):e0270875. doi: 10.1371/journal.pone.0270875 (PMC9779041; doi:10.1371/journal.pone.0270875)
Supplement: S2 Fig — Exemplary weekly documentation sheet of training sessions, selected exercises, assessment of pain and muscle soreness and additional exercise sessions apart from the intervention. The comment box allows participants to explain any deviation from the exercise selection and prescription. (PDF) [file pone.0270875.s003.pdf]

Training diary

Week 1

Session 1 - Date: _ _._ _ (Home ○ I Center ○)

Performed Exercises	A ○	B ○	C ○	D ○	E ○	F ○	G ○	H ○	I ○	J ○
Overall Perceived Exertion: Borg Scale (6-20) ______										
Muscle soreness (0-10)	Pain Score before training: ______ (0=No pain, 10=worst pain imaginable)					Location: Trunk ○ Legs ○ Other:___________				

Session 2 - Date: _ _._ _ (Home ○ I Center ○)

Performed Exercises	A ○	B ○	C ○	D ○	E ○	F ○	G ○	H ○	I ○	J ○
Overall Perceived Exertion: Borg Scale (6-20) ______										
Muscle soreness (0-10)	Pain Score before training: ______ (0=No pain, 10=worst pain imaginable)					Location: Trunk ○ Legs ○ Other:___________				

Session 3 - Date: _ _._ _ (Home ○ I Center ○)

Performed Exercises	A ○	B ○	C ○	D ○	E ○	F ○	G ○	H ○	I ○	J ○
Overall Perceived Exertion: Borg Scale (6-20) ______										
Muscle soreness (0-10)	Pain Score before training: ______ (0=No pain, 10=worst pain imaginable)					Location: Trunk ○ Legs ○ Other:___________				

Weekly summary

Additional training this week	Amount of training: __times à ___ Min<br>Type of training: endurance ○ strength ○ Other:_____________
Comments/ abnormalities of the week:	
