# Supplementary material for: Application of eccentric training in various clinical populations: Protocol for a multi-centered pilot and feasibility study in people with low back pain and people with multiple sclerosis
Source: PLoS One. 2022 Dec 22;17(12):e0270875. doi: 10.1371/journal.pone.0270875 (PMC9779041; doi:10.1371/journal.pone.0270875)
Supplement: S1 File — (PDF) [file pone.0270875.s004.pdf]

UNIVERSITÄT POTSDAM
- E T H I K K O M M I S S I O N -

Antrag (Auszug) an die Ethikkommission der Universität Potsdam
****

1. Antragsteller:

Institution:	Hochschulambulanz der Universität Potsdam,
Institut/Bereich an der UP:	Hochschulambulanz Universität Potsdam,<br>Professur für Sportmedizin und Sportorthopädie

2. Angaben zum Projekt:

Titel:	Anwendung von exzentrischem Training in der Therapie von Rückenschmerzen
Fachrichtung	Sportmedizin

3. Zielsetzung und Kurzbeschreibung des Projektes

Hintergrund und Problemstellung: Unspezifischer Rückenschmerz (low back pain, LBP) ist eine der Hauptursachen für die hohe Krankheitslast der Bevölkerung und lange Ausfallzeiten (Hartvigsen 2018). Die Prävalenz von LBP beträgt in den westlichen Industrieländern bis zu 50%, die Lebenszeit-Prävalenz sogar bis zu 90% (Airaksinen et al., 2006, Choi et al., 2010). Schmerzen und Muskelatrophie werden häufig von funktionellen Einschränkungen begleitet, die die Lebensqualität erheblich beeinflussen können. Körperliche Aktivität wird als fester Bestandteil der Standardbehandlung von LBP empfohlen (Choi et al., 2010). Dabei sollte der Fokus insbesondere auf der Kräftigung der Rumpfmuskulatur und der Verbesserung der neuromuskulären Kontrolle liegen, da ein Defizit dieser Parameter zur Entstehung von LBP-Symptomen beitragen kann.
Exzentrisches Training (muskelverlängernde Kontraktionen) führt zu einer größeren Steigerung der neuromuskulären Kapazität und zu einer stärkeren Verbesserung des Kraftzuwachses als vergleichbare Übungen, bei gleichzeitig geringerer kardiovaskulärer Belastung (Douglas et al., 2017). Neben reversible Muskelschäden und verzögertem Muskelkater kann in Folge exzentrischer Kontraktionen eine Immunreaktion ausgelöst werden (Peake et al., 2017), auf die eine entzündungshemmende Reaktion folgen kann. Zusammenfassend ist exzentrisches Training zum einen zeiteffizient, zum anderen auch für Patienten mit metabolischen und kardiovaskulären Begleiterkrankungen geeignet und daher für eine klinische Anwendung besonders vielversprechend. Es ist bereits bekannt, dass exzentrisches Training bei verschiedenen orthopädischen Pathologien wie Tendinopathien (Alfredson et al., 1998) und Arthrose (Vincent et al., 2019) zu einer Verbesserung der Funktion und Verringerung von Schmerzen führt. Obwohl exzentrisches Training besonders gut geeignet scheint, den Rumpf auf hohe Belastungen während abbremsender Bewegungen oder externer Störreize vorzubereiten, die während des täglichen Lebens wiederholt auftreten, wurde das exzentrische Training als therapeutischer Ansatz bei der Behandlung von LBP noch nicht umfassend untersucht.

Ziele der Studie: Ziel des Pilotprojektes ist es, die klinische Durchführbarkeit und Effizienz von alltagsnahem exzentrischen Training in der Therapie von Rückenschmerzen zu untersuchen. Dabei wird analysiert, ob eine Intervention mit exzentrischem Training zu ähnlichen Verbesserungen der Schmerzen und Funktionen bei LBP führt, wie dies bei anderen orthopädischen Pathologien bereits beschrieben wurde.

Studiendesign und Methodik: Die Wirksamkeit des exzentrischen Trainings als Intervention bei Rückenschmerzen wird durch Erfassung der subjektiven Schmerzwahrnehmung sowie durch Messung von Kraft, Funktion und Lebensqualität analysiert. Vor Beginn und nach der 6-wöchigen Trainingsintervention erfolgt jeweils eine Messung, die einen isokinetischen Krafttest, die Erfassung der Muskelaktivität, Funktions- und Mobilitätstests sowie Fragebögen zu subjektivem Schmerzempfinden, Rückenschmerz-bezogenen Funktionseinschränkungen und Lebensqualität umfasst. Das Trainingsprogramm besteht aus 3 Einheiten/ Woche (zwei center-based, eine home-based) und umfasst exzentrische Übungen, die auf die rumpf- und hüftumgreifende Muskulatur fokussiert sind. Während der Intervention wird nach jeder Trainingseinheit das subjektive Belastungsempfinden sowie der Muskelschmerz („Muskelkater") abgefragt. Zusätzlich werden zu verschiedenen Zeitpunkten Blutproben zur Erfassung spezifischer Marker des Muskelstoffwechsels und der Immunreaktion entnommen.

Probanden, Ein-/Ausschlusskriterien: Es werden Personen im Alter zwischen 18 und 65 Jahren mit wiederkehrenden Rückenschmerzen (Interventionsgruppe, N=15) eingeschlossen. Die Personen müssen von mindestens zwei Episoden (> 24h) Rückenschmerzen innerhalb der vergangenen 12 Monate berichten. Asymptomatische Personen werden der Kontrollgruppe (N=15) zugeordnet. Ausschlusskriterien sind: akute Infektion; Schwangerschaft; weitere Beschwerden/Erkrankungen, für die körperliche Aktivität kontraindiziert ist; persistierender Rückenschmerz zum Zeitpunkt der Studie (akut, innerhalb der letzten 7 Tage). Die Probandenrekrutierung erfolgt über die Hochschulambulanz der Universität Potsdam.

Eingesetzte Methoden und Risiken für Studienteilnehmer: Die Methoden der Anamnese, des Trainings- und Gesundheitsmonitorings und die klinischen Untersuchung stellen nach menschlichem Ermessen kein Risiko dar. Die Kraftleistungsfähigkeit wird durch dynamometergestützte Krafttests bestimmt. Diese weisen ein geringes Verletzungsrisiko (Muskel- und/oder Sehnenverletzung) auf, das über standardisiertes Aufwärmen, Positionieren auf dem Gerät und Gewöhnung an die einzelne Testbedingung minimiert werden kann. Die Messung der Muskelaktivität erfolgt durch Oberflächenelektromyographie (EMG). Diese Methode beinhaltet das Kleben von Einmalelektroden auf verschiedene Muskelbäuche der rumpfumgreifenden Muskulatur. Die Haut wird dabei rasiert, leicht angeraut, oberflächlich abgestorbene Hautschichten entfernt und schließlich mit Desinfektionsmittel (Entfernen von Fett) gereinigt. Hier ist keine Gefahr für den/die Probanden/Probandin gegeben. In sehr seltenen Fällen sind Hautunverträglichkeiten gegen das Elektrodengel beschrieben, die mit Wundsalbe behandelt werden. Die Blutentnahmen werden von medizinischem Fachpersonal durchgeführt. Es werden ca. 20 ml Blut in der Regel aus einer Vene in der Armbeuge für die Analyse verschiedener Blutparameter (u.a. Muskelstoffwechsel) entnommen. Außer einem kurzen Schmerz beim Einstich der Nadel kann es gelegentlich zu einer leichten Einblutung mit nachfolgendem Bluterguss („blauer Fleck") kommen, der innerhalb weniger Tage verschwindet. Um eine Kreislaufreaktion zu vermeiden, erfolgt die Blutentnahme im Liegen. Sollte dennoch eine Kreislaufreaktion auftreten, wird das Personal entsprechende Maßnahmen ergreifen (z.B. Hochlagern der Beine). Andere Risiken der Blutentnahme wie Infektion, Blutpfropfbildung (Thrombosierung) oder die Verletzung von benachbartem Gewebe und Nerven durch die Blutentnahmenadel sind sehr selten und bei geschultem Personal so gut wie ausgeschlossen. Die Messung der Standstabilität (sog. Posturale Kontrolle) wird auf einer stabilen flachen Kraftmessplatte im ein- und

beidbeinigen Stand durchgeführt. Bei einem möglichen Gleichgewichtsverlust ist das Absetzen des Fußes auf den Boden jederzeit möglich. Zudem wird die Messung jederzeit unmittelbar durch eine/n Mitarbeiter/in des Projektteams begleitet, sodass ein Festhalten bei Bedarf möglich ist. Die weiteren Funktions- und Mobilitätstests (Timed Up and Go, Chair Rise Test) umfassen Alltagsbewegungen, die nach ausführlicher Erklärung und unter Anleitung durchgeführt werden. Nach menschlichem Ermessen ist das Risiko einer Verletzung daher als sehr gering einzuschätzen. Die Trainingsintervention umfasst exzentrische Übungen, die selbstständig und ohne Großgeräte durchgeführt werden. Diese sind von Sportwissenschaftler/innen und Sporttherapeut/innen entwickelt und werden durch sie angeleitet und begleitet. Die Durchführung wird während des center-based Trainings immer von einem Trainer angeleitet. Des Weiteren wird das Training durch umfangreiche Materialien (z.B. schriftliche Anleitung, illustriert durch Bildtafeln; s. Anhang) unterstützt. Außerdem erfolgt eine regelmäßige Kontrolle der korrekten Ausführung durch Sportwissenschaftler/innen und Sporttherapeut/innen. Das Verletzungsrisiko ist bei diesem Aufbau der Trainingsintervention als gering einzuschätzen. Infolge des Trainings kann starker Muskelkater auftreten, der nach wenigen Tagen verschwindet und mit ansteigender Trainingsdauer aufgrund der körperlichen Anpassung reduziert wird.

Datenerfassung und Datenschutz: Personenbezogene Daten der Studienteilnehmer/innen werden nach Studieneinschluss einmalig auf einem gesonderten Deckblatt erfasst, wenn das schriftliche Einverständnis vorliegt. Die erhobenen Daten werden psydonymisiert. Die Archivierung, Speicherung und Dokumentation der erhobenen Daten erfolgt auf passwortgeschützten, abteilungsinternen Computern sowie anhand eines webbasierten Dokumentationsformulars entsprechend der geltenden datenschutzrechtlichen Bestimmungen (Datenschutzgrundverordnung). Alle Studienunterlagen werden stets unter Verschluss gehalten und im Anschluss an Auswertung, wissenschaftliche Aufarbeitung und Publikationen nach der vorgeschriebenen Aufbewahrungsfrist (10 Jahre) wieder vernichtet bzw. gelöscht. Die an der Studie beteiligten Ärzt/innen, Sporttherapeut/innen sowie das weitere medizinische und wissenschaftliche Personal unterliegen der gesetzlichen Schweigepflicht und werden auf das Datengeheimnis verpflichtet.

Referenzen

Airaksinen O, Brox JI, Cedraschi C, Hildebrandt J, Klaber-Moffett J, Kovacs F, Mannion AF, Reis S, Staal JB, Ursin H, Zanoli G (2006). Chapter 4. European guidelines for the management of chronic nonspecific low back pain. Eur Spine J. 2006; 2: S192-300.

Alfredson H, Pietilä T, Jonsson P, Lorentzon R. Heavy-load eccentric calf muscle training for the treatment of chronic Achilles tendinosis. Am J Sport Med 1998; 26: 360–366.

Choi BK, Verbeek JH, Tam WW, Jiang JY (2010). Exercises for prevention of recurrences of low-back pain. Cochrane Database Syst Rev. 2010; 1:CD006555.

Douglas J, Pearson S, Ross A, McGuigan M. Chronic Adaptations to Eccentric Training: A Systematic Review. Sports Med 2017; 47: 917–941.

Hartvigsen J, Hancock MJ, Kongsted A, Louw Q, Ferreira ML, Genevay S et al. What low back pain is and why we need to pay attention. Lancet 2018; 391(10137):2356–67.

Peake JM, Neubauer O, Della Gatta PA, Nosaka K (2017). Muscle damage and inflammation during recovery from exercise. J Appl Physiol (1985). 2017 Mar 1;122(3):559-570.

Vincent KR, Vasilopoulos T, Montero C, Vincent HK. Eccentric and Concentric Resistance Exercise Comparison for Knee Osteoarthritis. Med Sci Sport Exerc 2019; 1.
