# Supplementary material for: Application of eccentric training in various clinical populations: Protocol for a multi-centered pilot and feasibility study in people with low back pain and people with multiple sclerosis
Source: PLoS One. 2022 Dec 22;17(12):e0270875. doi: 10.1371/journal.pone.0270875 (PMC9779041; doi:10.1371/journal.pone.0270875)
Supplement: S2 File — (PDF) [file pone.0270875.s005.pdf]

UNIVERSITÄT POTSDAM
- E T H I K K O M M I S S I O N -

Ethics proposal (excerpt) to the ethics committee of the University of Potsdam
****

**1. Applicant:**	
Institution:	University Outpatient Clinic, Potsdam
Institute/Department at the UP:	University Outpatient Clinic,<br>Professorship of Sports Medicine and Sports Orthopaedics
**2. Project Information:**	
Title:	Application of eccentric training in the therapy of low back pain
Subject area:	Sports Medicine

3. Purpose, Short Description and Classification of the Project

Background: Nonspecific low back pain (low back pain, LBP) is one of the main causes of the high disease burden in the population and long periods of absence (Hartvigsen 2018). The prevalence of LBP in the western industrialized countries is up to 50%, the lifetime prevalence even up to 90% (Airaksinen et al., 2006, Choi et al., 2010). Pain and muscle atrophy are often accompanied by functional limitations that can have a significant impact on the quality of life. Physical activity is recommended as an integral part of standard treatment for LBP (Choi et al., 2010). The focus should be on strengthening the core muscles and improving neuromuscular control, as a deficit of these parameters can contribute to the development of LBP symptoms.
Eccentric training (muscle-lengthening contractions) leads to a greater increase in neuromuscular capacity and a greater improvement in strength gain than comparable exercises, while at the same time causes less cardiovascular stress (Douglas et al., 2017). In addition to reversible muscle damage and delayed muscle soreness, eccentric contractions can trigger an immune reaction (Peake et al., 2017), which can be followed by an anti-inflammatory reaction. In summary, eccentric training is on the one hand time-efficient, on the other hand it is also suitable for patients with metabolic and cardiovascular concomitant diseases and is therefore particularly promising for clinical use. It is already known that eccentric training leads to an improvement in function and a reduction in pain in various orthopedic pathologies such as tendinopathies (Alfredson et al., 1998) and osteoarthritis (Vincent et al., 2019). Although eccentric training seems to be particularly well suited to prepare the trunk for high loads during external disturbances that occur repeatedly during everyday life, eccentric training has not yet been extensively investigated as a therapeutic approach in the treatment of LBP.
Purpose: The aim of the pilot project is to examine the clinical feasibility and efficiency of everyday eccentric training in the therapy of back pain. It is analyzed whether an intervention with eccentric training leads to similar improvements in pain and functions in LBP as has already been described for other orthopedic pathologies.
Study design and methods: The effectiveness of eccentric training as an intervention in low back pain is analyzed by recording the subjective pain perception as well as by measuring strength, function and quality of life. Before and after the 6-week training intervention, measurements are

performed including isokinetic strength tests, muscle activity assessments, function and mobility tests, and questionnaires on subjective pain perception, functional limitations related to back pain and quality of life. The training program consists of 3 sessions / week (two center-based, one home-based) and includes eccentric exercises that focus on trunk and hip encompassing muscles. For each training session during the intervention subjective rating of perceived exertion and muscle soreness are assessed. In addition, blood samples are taken at various times to record specific markers of muscle metabolism and the immune reaction.

Participants, Inclusion criteria/Exclusion criteria: People between the ages of 18 and 65 with recurring back pain (intervention group, N = 15) are included. People must report at least two episodes (> 24h) of back pain within the past 12 months. Asymptomatic persons are assigned to the control group (N = 15). Exclusion criteria are acute infections; pregnancy; other complaints / illnesses for which physical activity is contraindicated; persistent back pain at the time of the study (acute, within the last 7 days). The eligible participants are recruited via the university outpatient clinic of the University of Potsdam.

Applied methods and risks for participants: Anamnesis, training and health monitoring and the clinical examination do not represent a risk as far as humanly possible. The strength performance is determined by dynamometer-based strength tests. These show a low risk of injury (muscle and / or tendon injury), which is minimized by a standardized warm-up, positioning on the device and getting used to the individual test conditions. Muscle activity is measured using surface electromyography (EMG). This method involves attaching disposable electrodes on various muscle bellies of the trunk encompassing musculature. The skin is shaved, slightly abraded to remove dead skin layers from the surface and finally cleaned with disinfectant (removal of fat). There is no danger for the test person here. In very rare cases, skin intolerance to the electrode gel has been described, which is treated with ointment. The blood sampling are carried out by healthcare professionals. Approx. 20 ml of blood is usually taken from a vein in the crook of the arm for the analysis of various blood parameters (including muscle metabolism). In addition to a brief pain when the needle is inserted, there may occasionally be a slight bleeding followed by a bruise, which will vanish within a few days. In order to avoid a circulatory reaction, the blood is drawn while lying down. Should a circulatory reaction nevertheless occur, the staff will take appropriate measures (e.g. elevating the legs). Other risks of blood collection such as infection, blood clot formation (thrombosis) or damage to neighboring tissue and nerves by the needle are very rare and practically impossible with trained personnel. The measurement of the stance stability (so-called postural control) is carried out on a stable, flat force plate with one and two-legged stance. If balance is lost, it can be regained by putting down the foot at any time point. In addition, the measurement is directly accompanied by a member of the research team to support stance stability. The other function and mobility tests (Timed Up and Go, Chair Rise Test) include everyday movements that are carried out after a detailed explanation and under guidance. The risk of injury can therefore be assessed as very low. The training intervention includes eccentric exercises that are carried out independently and without large equipment. These have been developed by sports scientists and sports therapists and are instructed and accompanied by them. The implementation is always guided by a therapist during the center-based training. Furthermore, the training is supported by extensive materials (e.g. written instructions, illustrated by picture boards). In addition, the correct execution is regularly checked by sports scientists and sports therapists. The risk of injury is estimated low with this structure of the training intervention. As a result of the training, severe muscle soreness can occur, which disappears after a few days and is reduced with increasing training duration due to the physical adaptation.

Data Collection, Safety and Protection: With written consent given, personal data of the study participants are recorded once on a separate cover sheet after inclusion in the study. The collected data are pseudonymized. The archiving, storage and documentation of the collected data takes place on password-protected, department-internal computers as well as on a web-based documentation form in accordance with the applicable data protection regulations (Datenschutzgrundverordnung). All study documents are always kept locked. Following data

analysis, result synthesis and publication of scientific findings, all study documents will be destroyed or deleted after the prescribed retention period (10 years). Physicians, sports therapists and other medical and scientific staff involved in the study are subject to statutory confidentiality and are obliged to maintain data secrecy.

References

Airaksinen O, Brox JI, Cedraschi C, Hildebrandt J, Klaber-Moffett J, Kovacs F, Mannion AF, Reis S, Staal JB, Ursin H, Zanoli G (2006). Chapter 4. European guidelines for the management of chronic nonspecific low back pain. Eur Spine J. 2006; 2: S192-300.

Alfredson H, Pietilä T, Jonsson P, Lorentzon R. Heavy-load eccentric calf muscle training for the treatment of chronic Achilles tendinosis. Am J Sport Med 1998; 26: 360–366.

Choi BK, Verbeek JH, Tam WW, Jiang JY (2010). Exercises for prevention of recurrences of low-back pain. Cochrane Database Syst Rev. 2010; 1:CD006555.

Douglas J, Pearson S, Ross A, McGuigan M. Chronic Adaptations to Eccentric Training: A Systematic Review. Sports Med 2017; 47: 917–941.

Hartvigsen J, Hancock MJ, Kongsted A, Louw Q, Ferreira ML, Genevay S et al. What low back pain is and why we need to pay attention. Lancet 2018; 391(10137):2356–67.

Peake JM, Neubauer O, Della Gatta PA, Nosaka K (2017). Muscle damage and inflammation during recovery from exercise. J Appl Physiol (1985). 2017 Mar 1;122(3):559-570.

Vincent KR, Vasilopoulos T, Montero C, Vincent HK. Eccentric and Concentric Resistance Exercise Comparison for Knee Osteoarthritis. Med Sci Sport Exerc 2019; 1.
