# Supplementary material for: Application of eccentric training in various clinical populations: Protocol for a multi-centered pilot and feasibility study in people with low back pain and people with multiple sclerosis
Source: PLoS One. 2022 Dec 22;17(12):e0270875. doi: 10.1371/journal.pone.0270875 (PMC9779041; doi:10.1371/journal.pone.0270875)
Supplement: S3 File — (PDF) [file pone.0270875.s006.pdf]

Ethics proposal (excerpt) – Chaim Sheba Medical Center

1. Introduction

Eccentric training (ECC) is known to lead to improvement of function and reduction of pain in context of various pathologies/structures, such as tendinopathies [1,2], prevention of hamstring injuries [3], arthrosis [4] and chronic obstructive pulmonary disease [5]. From a clinical perspective, ECC appears to particularly be beneficial because of its efficiency regarding superior strength gains, strong everyday relevance, time efficiency, lower metabolic demands and possible post-exercise anti-inflammatory reactions [6]. Accordingly, the clinical application of ECC seems to be promising in neurologic diseases, such as Multiple Sclerosis (MS) [7]. However, only few small studies examined this treatment paradigm in the MS population. Therefore, the purpose of the present pilot study is to investigate the clinical feasibility and efficiency of ECC for people with MS.

2. Materials and Methods

2.1. Study design

- Intervention pilot study
- implication of ECC training

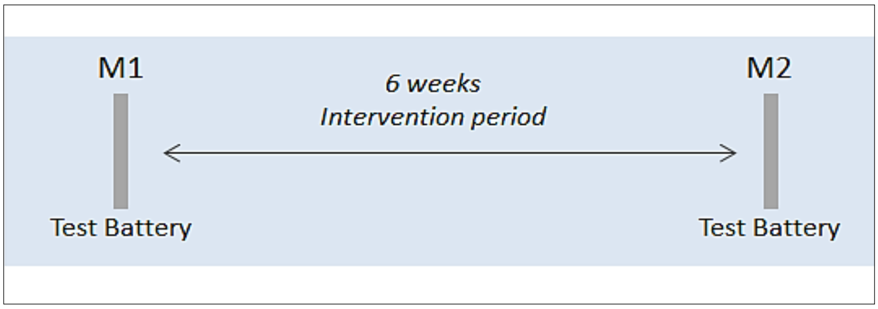

2.2 Subjects

<u>I. People with Multiple Sclerosis</u> (N = 24)

- Inclusion criteria:
  - A neurologist-confirmed diagnosis of definite MS according to the revised McDonald criteria Exclusion criteria [8]
  - <4.5 score on the Expanded Disability Status Scale (EDSS) [9], the ability to walk an equivalent of at least 500m without a walking aid
  - Relapse-free for at least 90 days prior to testing
  - Males and females 18-65 years old
- Exclusion criteria:
  - Pregnancy
  - Cardiovascular disorders
  - Respiratory disorders
  - Taking steroids or fampridine

- Recruitment:
  - Multiple Sclerosis Center, Sheba Medical Center, Tel-Hashomer, Israel.

2.3 Assessment of mobility, pain and physical related functions

All tests will be measured at M1 and M2

a. Physical examination

- Screening: contraindications for following motor function assessment and intervention

b. Self-report questionnaires

- SF 36 quality of life in MS [10, 11]
- Multiple Sclerosis Walking Scale (MSWS-12) [12]
- Modified Fatigue Impact Scale (MFIS) [13]
- Falls Efficacy Scale International (FES-I) [14]

c. Strength capacity

- isokinetic dynamometry - Leg strength (Biodex), unilateral (both legs) knee extension/flexion
  → ROM: 90° to 5° flexion

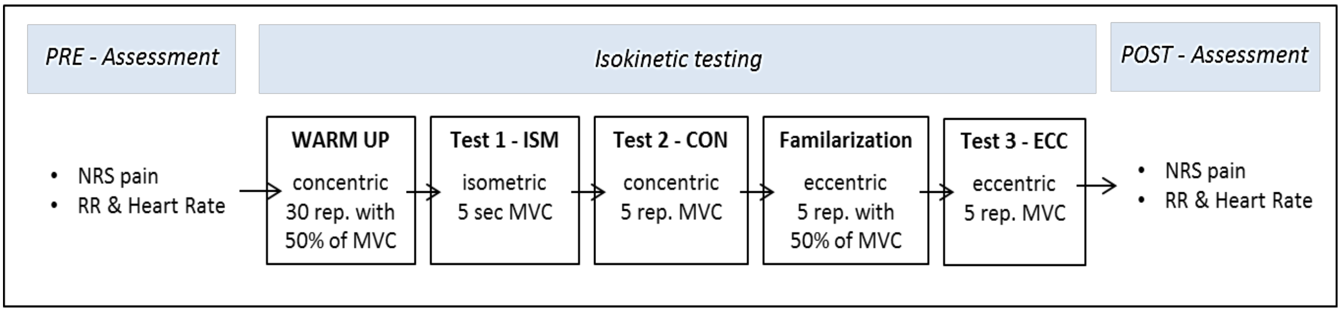

d. Function/Mobility

- Timed Up and Go (TUG) [15, 16] → mobility testing MS [7, 17, 18]
- Chair rise test (CR/5R-STS) [19] → lower extremity power and mobility in MS [20]
- Two-minute Walk Test [21]

e. Postural Control

- Single-leg stance over 30 sec (sway of COP) [22]
  - assessment with Wii Balance boards (CSMI solutions)
- optional: Functional Reach Test (FRT) [23, 24]
- optional: accelerometer system (APDM) [25] (balance)
- optional: Four Step Square test (FSST) [26]

2.4. Intervention

General Principle

➔ Focused trunk and hip exercises relevant for everyday loading situations [See training booklet]

- accentuated eccentric training [27]: slow movement execution during eccentric phase – fast/supported concentric movement back to starting position

Design

- eccentric training instead of "usual care"
- combined center-(2x/wk) & home-based (1x/wk) = 18 sessions within 6 weeks
- catalogue of 10 exercises for both pathologies – 4 exercises each session (2 trunk, 2 hip)
- multiple sets of exercises (3x10-15 rep.)

Dose/intensity control

- duration per session: 45 – 60 min
- control of individual intensity: via BORG scale [7], e.g. between level 12 to 15 (somewhat hard to hard)
- assessment of pain: NRS before and after each training session
- possible progression [7, 28]:
    - consideration of repeated bout effect by moderate progression of intensity [29]
    - increase of sets, e.g. from 1 to 3 within first 3 weeks
    - increase of lever-arm and load (dumb bell, Thera-band) during eccentric phase

Assessment of Muscle Damage / Inflammation markers

- Overall 3 measurement time points:

1) before M1 (pre, baseline);
2) 0.5h after the first training session;
3) 72 h after last training session = before M2;

Blood sampling (5cc), centrifugation → serum samples (multiple aliquots per subject & time point) → freezing → storage at – 70 °C

<u>Analysis</u>: Parameters of interest: creatine kinase (CK), Interleukin-6 (IL-6), Interleukin-10 (IL-10) [Optional: Tumor necrosis factor α (TNFα), Interleukin-1ra (IL-1ra)].

References

[1] Camargo PR. Eccentric training as a new approach for rotator cuff tendinopathy: Review and perspectives. *World J Orthop* 2014; 5: 634.

[2] Malliaras P, Barton CJ, Reeves ND, et al. Achilles and Patellar Tendinopathy Loading Programmes. *Sport Med* 2013; 43: 267–286.

[3] Shadle IB, Cacolice PA. Eccentric Exercises Reduce Hamstring Strains in Elite Adult Male Soccer Players: A Critically Appraised Topic. *J Sport Rehabil* 2017; 26: 573–577.

[4] Gür H, Çakin N, Akova B, et al. Concentric versus combined concentric-eccentric isokinetic training: Effects on functional capacity and symptoms in patients with osteoarthrosis of the knee. *Arch Phys Med Rehabil* 2002; 83: 308–316.

[5] MacMillan NJ, Kapchinsky S, Konokhova Y, et al. Eccentric ergometer training promotes locomotor muscle strength but not mitochondrial adaptation in patients with severe chronic obstructive pulmonary disease. *Front Physiol* 2017; 8: 1–14.

[6] Hedayatpour N, Falla D. Physiological and Neural Adaptations to Eccentric Exercise: Mechanisms and Considerations for Training. *Biomed Res Int* 2015; 2015: 193741.

[7] Hayes HA, Gappmaier E, Lastayo PC. Effects of high-intensity resistance training on strength, mobility, balance, and fatigue in individuals with multiple sclerosis: A randomized controlled trial. *J Neurol Phys Ther* 2011; 35: 2–10.

[8] Polman CH, Reingold SC, Banwell B, et al. Diagnostic criteria for multiple sclerosis: 2010 revisions to the McDonald criteria. *Ann Neurol* 2011; 69: 292–302.

[9] Kurtzke JF. Rating neurologic impairment in multiple sclerosis: an expanded disability status scale (EDSS). *Neurology* 1983; 33: 1444–52.

[11] Ware JE, Sherbourne CD. The MOS 36-item short-form health survey (SF-36). I. Conceptual framework and item selection. *Med Care* 1992; 30: 473–83.

[12] Riazi A, Hobart JC, Lamping DL, et al. Using the SF-36 measure to compare the health impact of multiple sclerosis and Parkinson's disease with normal population health profiles. *J Neurol Neurosurg Psychiatry* 2003; 74: 710–4.

[14] McGuigan C, Hutchinson M. Confirming the validity and responsiveness of the Multiple Sclerosis Walking Scale-12 (MSWS-12). *Neurology* 2004; 62: 2103–5.

[15] Learmonth YC, Dlugonski D, Pilutti LA, et al. Psychometric properties of the Fatigue Severity Scale and the Modified Fatigue Impact Scale. *J Neurol Sci* 2013; 331: 102–7.

[16] van Vliet R, Hoang P, Lord S, et al. Falls efficacy scale-international: a cross-sectional validation in people with multiple sclerosis. *Arch Phys Med Rehabil* 2013; 94: 883–9.

[17] Schoene D, Wu SM-S, Mikolaizak AS, et al. Discriminative Ability and Predictive Validity of the Timed Up and Go Test in Identifying Older People Who Fall: Systematic Review and Meta-Analysis. *J Am Geriatr Soc* 2013; 61: 202–208.

[18] Podsiadlo D, Richardson S. The timed 'Up & Go': a test of basic functional mobility for frail elderly persons. *J Am Geriatr Soc* 1991; 39: 142–8.

[19] Kalron A, Dolev M, Givon U. Further construct validity of the Timed Up-and-Go Test as a measure of ambulation in multiple sclerosis patients. *Eur J Phys Rehabil Med* 2017; 53: 841–847.

[20] Cattaneo D, Regola A, Meotti M. Validity of six balance disorders scales in persons with multiple sclerosis. *Disabil Rehabil* 2006; 28: 789–795.

[22] Guralnik JM, Simonsick EM, Ferrucci L, et al. A short physical performance battery assessing lower extremity function: association with self-reported disability and prediction of mortality and nursing home admission. *J Gerontol* 1994; 49: M85-94.

[23] Romberg A, Ikonen A, Ruutiainen J, et al. The effects of heat stress on physical functioning in persons with multiple sclerosis. *J Neurol Sci* 2012; 319: 42–6.

[25] Baert I, Freeman J, Smedal T, et al. Responsiveness and Clinically Meaningful Improvement, According to Disability Level, of Five Walking Measures After Rehabilitation in Multiple Sclerosis. *Neurorehabil Neural Repair* 2014; 28: 621–631.

[26] Clark RA, Mentiplay BF, Pua YH, et al. Reliability and validity of the Wii Balance Board for assessment of standing balance: A systematic review. *Gait Posture* 2018; 61: 40–54.

[27] Duncan PW, Weiner DK, Chandler J, et al. Functional reach: a new clinical measure of balance. *J Gerontol* 1990; 45: M192-7.

[28] Pieber K, Herceg M, Csapo R, et al. Effects of a multidisciplinary programme on postural stability in patients with chronic recurrent low back pain: preliminary findings. *Eur Spine J* 2016; 25: 1219–1225.

[29] Grinberg Y, Berkowitz S, Hershkovitz L, et al. The ability of the instrumented tandem walking tests to discriminate fully ambulatory people with MS from healthy adults. *Gait Posture* 2019; 70: 90–94.

[30] Kalron A, Givon U. Construct Validity of the Four Square Step Test in Multiple Sclerosis. *Arch Phys Med Rehabil* 2016; 97: 1496–1501.

[31] Suchomel TJ, Nimphius S, Stone MH. The Importance of Muscular Strength in Athletic Performance: Training Considerations. *Sport Med* 2018; PUB AHEAD: 1–21.

[32] Kraemer WJ, Ratamess NA. Fundamentals of resistance training: progression and exercise prescription. *Med Sci Sports Exerc* 2004; 36: 674–88.

[33] Bridgeman LA, Mcguigan MR, Gill ND. Eccentric exercise, exercise induced muscle damage and the repeated bout effect: A brief revie. *J Aust Strength Cond* 2015; 23: 74–84.
